# Diseases of the Kidney

**Published:** 1901-11-09

**Authors:** 


### Diseases of the Kidney.

(*Continued from page* 85.)

**Arterio-sclerosis.** — Herringham[10] discusses the relation between endarteritis and Bright's disease and holds that the two are almost independent. Arterial degeneration if caused by kidney trouble is rather due to the general failure of nutrition than to obstruction or chemical irritation of the vessels. The age of the patient is a chief factor in its production. The high-tension pulse is due to the failure of the elasticity of the larger vessels rather than to peripheral obstruction. In 34 cases of Bright's disease he found no instance of true hypertrophy or hyperplasia of the muscular tissue of the renal arterioles, while in the larger vessels there was increase of the elastic tissue. W. Baxter Gow[11] has investigated the relation between chronic renal disease and the conditions of excitement and depression in the insane from 100 post-mortems in the Lancashire Asylum. It appears from various statistics that the frequency of granular kidney in the insane is about 45 per cent., or 20 per cent. more than in ordinary persons; yet the ordinary reputed causes of the disease are not more common in the insane than in the sane. The writer finds that the frequency is connected with the mental condition and especially with the presence of motor excitement, whereas in melancholia and delusional states the kidneys are more often normal. Granular kidneys again do not usually produce their ordinary symptoms when present in the insane, and indeed the blood pressure is generally low in the type of insanity in which they are commonly found. Thus, too, there is rarely cardiac hypertrophy, albuminuria, excess in the amount of urine or the signs of uræmia. The low blood pressure in the states marked by excitement seems to be in some way connected with the causation of granular kidney. W. Russell[12] points out that in this country the theory of arterio-sclerosis most frequently adopted is that the thickening of the intima is secondary to a dilatation of the vessels and slowing of the blood stream, and indeed is an attempt at compensation which restores its rapidity. From the microscopical examination of the vessels in 16 cases he shows that this view is without foundation, and that error has arisen from not noting the difference of the changes in the arteries outside and inside the kidneys. The former showed a true hypertrophy of the muscular coat, while in the kidneys there is usually none, though the intima is much more thickened than elsewhere. The changes in the vessels of the brain correspond with those in the kidney. Thus the condition of the radial artery is no index of that of the vessels in the brain or kidney, and it is also important to distinguish between thickening of vessels and hypertonus or continued contraction of them. This latter condition is generally due to the presence of some poison in the blood. How then are arterio-sclerosis and hypertonus connected? He thinks that continued contraction leads to hypertrophy of the muscular coat, while the continued presence of poisons leads to fibrous hyperplasia of the intima. The nephritis of pregnancy is shown by Gaucher and Sergent[13] to correspond to the evolution of interstitial nephritis in other toxic cases. A single pregnancy with auto-intoxication may cause acute nephritis from hepatic toxæmia, while repeated ones have the same effect as slow prolonged intoxications, and tend to produce interstitial changes. Rose Bradford's remarks[14] on the clinical varieties of Bright's disease are worthy of notice. Besides true granular kidney disease, he recognises two forms of acute Bright's disease. In one there are the urinary symptoms with dropsy, and in the other, which shades off into mere febrile congestion of the kidney, dropsy is absent. There are also two chronic forms, one with dropsy and scanty albuminous urine, and the other with no dropsy and often with no symptoms, or only wasting malaise and copious albuminous urine, until fatal uræmia sets in. The last form is due to a granular small kidney with shrunken cortex, while in the dropsical form we find a kidney of normal or rather increased size, with a thickened cortex and smooth surface when peeled.

10 Lancet, Feb. 23. 11 New Zealand Med. Jour., Feb. 12 Brit. Med. Jour., May, 18. 13 Med. Chron., April. 14 Lancet, Jan. 5.

